# Use of a neuro-evacuation device for the endoscopic removal of third ventricle colloid cysts

**DOI:** 10.3389/fsurg.2023.1214290

**Published:** 2023-07-11

**Authors:** Stefano Peron, Nicola Galante, Donato Creatura, Giovanni Marco Sicuri, Roberto Stefini

**Affiliations:** ^1^ Department of Neurosurgery, ASST West Milan—Legnano Hospital, Legnano (Milan), Italy; ^2^ Department of Neurosurgery, IRCCS Humanitas Research Hospital, Milan, Italy

**Keywords:** Artemis, colloid cyst, minimally invasive surgery, neuroendoscopy, endoscopic transforaminal approach

## Abstract

**Background:**

Colloid cysts are benign tumors usually located at the level of the foramen of Monro and account for approximately 1% of all intracranial tumors. Endoscopic surgical treatment represents the approach of choice for removal of these tumors and is usually preferred over transcortical or transcallosal microsurgical approaches. Our purpose is to demonstrate the feasibility of endoscopic removal of colloid cysts using a novel aspiration and fragmentation system, currently designed for evacuation of cerebral hematomas.

**Methods:**

We performed an evaluation of the results obtained in patients with symptomatic colloid cysts of the third ventricle operated on using an endoscopic neuroevacuation system (Artemis Neuro Evacuation Device, Penumbra, Alameda, California, USA) between April 2020 and April 2022. Instrumentation and surgical technique are described in detail. All patients underwent postoperative MRI to assess the extent of cyst removal.

**Results:**

Five patients were included in our study. The predominant symptom at onset was headache. No intraoperative complications related to the technology in use occurred. The surgical time for the cyst removal was significantly shorter than removal via a standard endoscopic technique (80 vs. 120 min). Removal was complete, both content and capsule of the cyst, in all patients. In all cases there was a complete regression of the previously complained symptoms.

**Conclusion:**

The Artemis Neuro Evacuation Device has proved to be effective and safe in removal of colloid cysts of the third ventricle and may be proposed as a possible alternative or as a complement of the standard instruments routinely used in neuroendoscopy.

## 1. Introduction

Colloid cysts of the third ventricle account for 1%–2% of all intracranial tumors, arising from the roof of the anterior third ventricle with a wall of ciliated epithelial cells and mucin content (1–3). In asymptomatic patients, observation could be an option (4–6). However, due to their location close to one or both foramina of Monro, obstruction of cerebrospinal fluid (CSF) flow may result in ventriculomegaly and hydrocephalus, causing different symptoms (1, 4, 6).

Surgical removal is the preferred treatment for symptomatic patients with a colloid cyst of the third ventricle. In the past, microsurgical resection through a transcallosal or transcortical approach has been the most common approach for removal of these lesions (2–6). In the last few decades, the minimally invasive endoscopic removal of the cysts has been shown to be safe with remarkable advantages in terms of length of hospital stay, postoperative pain and surgical morbidity (3–5). However, surgical success is strongly related to the endoscopic instrumentation available.

More recently, a novel aspiration and fragmentation system (Artemis Neuro Evacuation Device, Penumbra, Alameda, California, USA) has been designed for minimally invasive endoscopic evacuation of intracerebral hemorrhages. Indeed, this system takes advantage of standard cranial neuroendoscopy concepts and applies them to the removal of intracerebral hematomas, such as for Stereotactic Intracerebral Hemorrhage Underwater Blood Aspiration (SCUBA) technique. We considered these characteristics appropriate even for endoscopic removal of colloid cysts.

The purpose of this study is to assess the safety and feasibility of using this endoscopic neuroevacuation system for the removal of colloid cysts of the third ventricle.

## 2. Material and methods

### 2.1. Aspiration and fragmentation system

The Artemis Device is a single-use straight device with diameters from 1.5 to 2.8 mm. The handle of the device is connected to a vacuum pump via flexible tubing. A maximum pressure of 29 in Hg (98.2 kPa) with a flow rate of 0.8 standard cubic feet per minute can be generated, while a knob on the pump permits a fine-controlling aspiration.

This system provides powerful and controlled aspiration that, together with its built-in fragmentation mechanism, allows a safe, gentle, precise, and constant removal of the blood clot. All the components of the system are physician controllable. In this way, if the surgeon notices that an artery or a vein are in the path of the tip, suction pressure can be easily adjusted to minimize the risk of iatrogenic vascular injury, both to the thalamostriate vein and the choroid plexus.

**Figure 1:**
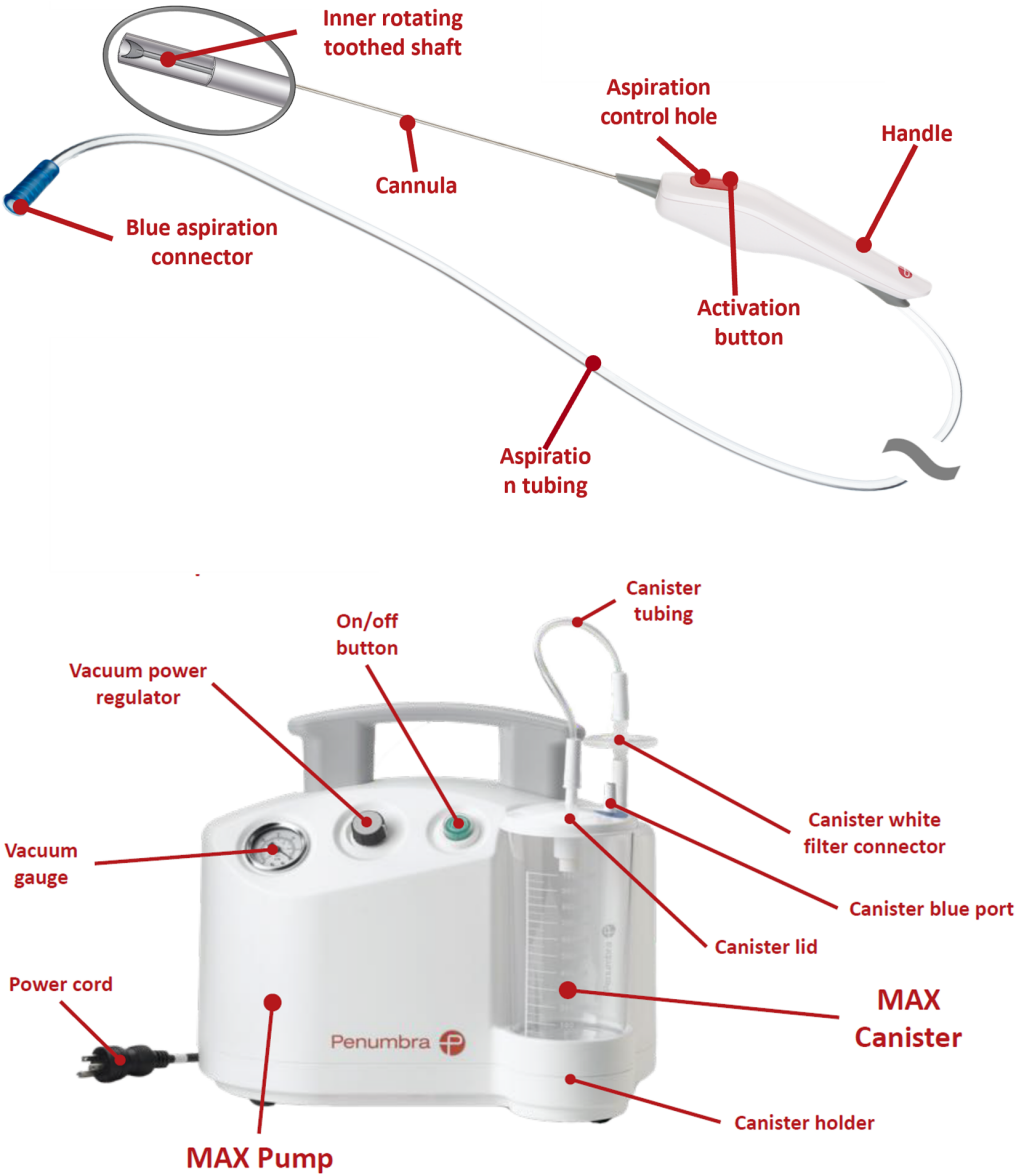
The artemis neuro evacuation device; to note, the activation button with aspiration control hole on the handle.

The hematoma must extrude into the tip of the device cannula, under aspiration, before the fragmentation mechanism, an inner rotating toothed shaft can act. The high-torque rotation of that inner shaft allows the clot fragmentation, only inside the lumen of the cannula, for a continuous aspiration without clogging. All technical details about this system are summarized in Figure 1.

### 2.2. Operative technique

We retrospectively evaluated 5 patients operated on for colloid cysts of the third ventricle with an endoscopic approach associated with the use of the Artemis Device between April 2020 and April 2022. Institutional review board approved the study and a written consent was collected from all patients undergoing the procedure. All patients underwent preoperative imaging by contrast-enhanced MRI.

The surgical procedure involved a purely endoscopic transforaminal approach, following the standard technique to access the lateral ventricle through Kocher's point. The access varied between the right or left sides depending on the location of the cyst relative to the roof of the third ventricle and the size of the foramina of Monro and the lateral ventricles.

Through a small incision in the frontal region, a precoronaric burr hole was created and a 19 French introducer peel-away sheath was inserted to access the lateral ventricle. Then, a Storz 6-degree viewing angle rigid LOTTA Neuroendoscope (Karl Storz, Tuttlingen, Germany) or, alternatively, an Aesculap 0-degree rigid MINOP Neuroendoscope (B. Braun—Aesculap, Tuttlingen, Germany), was inserted.

**Figure 2:**
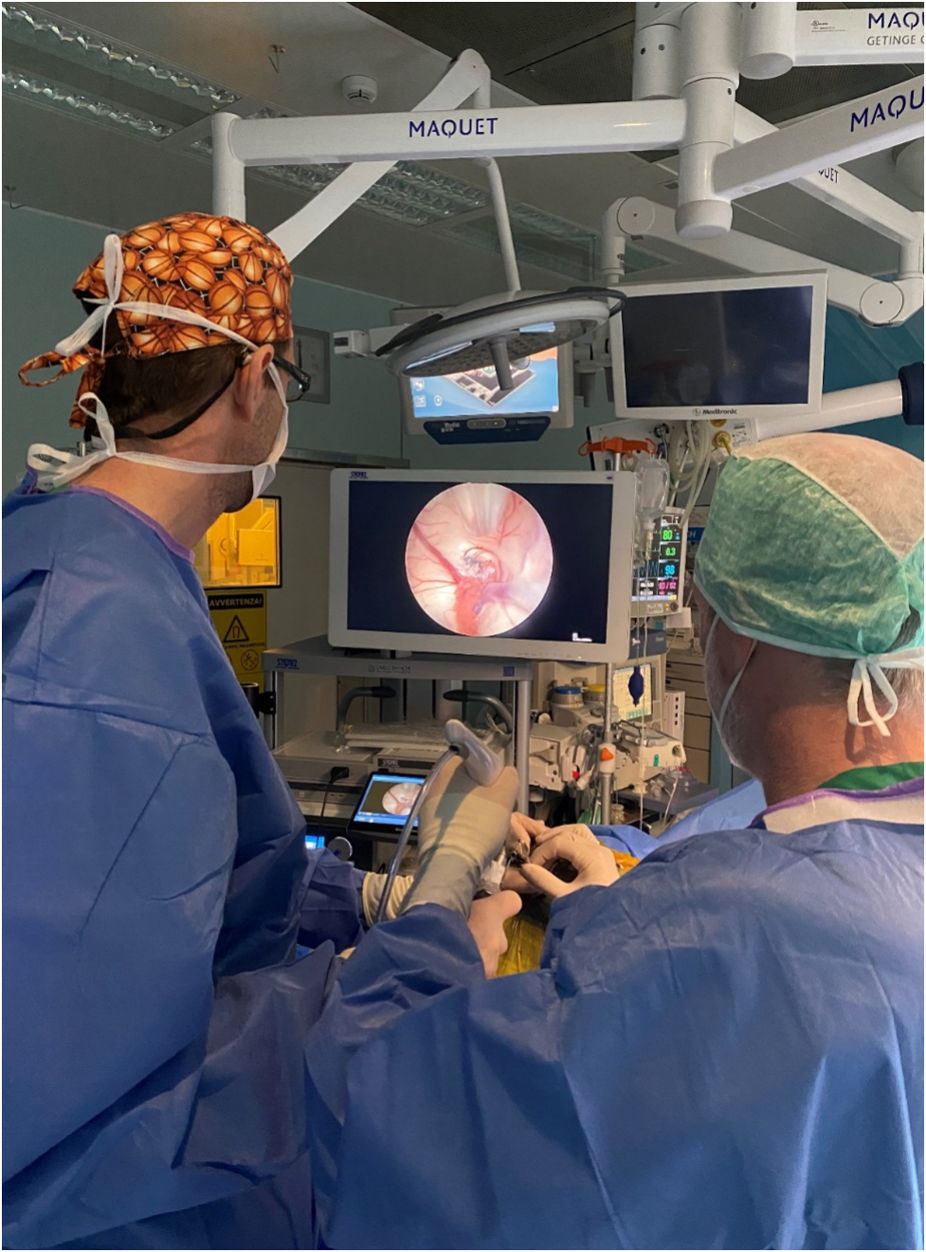
The 2.8 mm cannula of the artemis device is introduced into the ventricle through the working channel of a rigid neuroendoscope.

Once the cyst was in view, a 2.8 mm or 2.1 mm Artemis Device was introduced into the ventricle through the working channel of the rigid endoscope (Figure 2). In our cases, removal of the cyst was achieved by fragmentation of the capsule and aspiration of its mucinous contents with the Artemis Device. Continuous irrigation was used to enhance anatomic visualization and control any venous bleeding from the choroid plexus. Bipolar coagulation was applied when necessary. Additionally, sampling of the cyst capsule was performed, and the aspiration reservoir allowed for obtaining more material for histological examination.

All procedures were performed by experienced senior surgeons with expertise in endoscopic ventricular surgery. At the conclusion of the surgery, the extent of cyst removal, including the capsule and intracystic content, was evaluated.

A postoperative MRI was obtained within 72 h after surgery and 3 months later, to confirm the absence of residual cysts and assess ventricular size.

## 3. Results

In this study, we treated 5 patients, who had colloid cysts of the third ventricle, via an endoscopic surgical approach assisted by the Artemis Device. The patients, all female, had a mean age of 45 years (range 28–65).

In all cases, the main symptom at onset was headache, with two patients also complaining of nausea and vomiting, one patient with short-term memory impairment and difficulty concentrating at work, and one patient with gait disturbances.

All patients came to our outpatient clinic with a contrast-enhanced brain MRI, as prescribed by their primary care physician or neurologist. The average cyst size was 12 mm (range 9–15), and hydrocephalus or ventriculomegaly was observed in 4 out of 5 patients. Although occasionally the contents of the cyst may be firm, in all our cases the contents were soft.

The average duration of the procedure was approximately 80 min, representing an important reduction in operative time compared to the average of 120 min required for the previous five colloid cyst removals performed using standard endoscopic techniques.

In all cases, the entire procedure of removing the cyst and its contents was performed with the Artemis, without the need to use grasping forceps, scissors or other conventional instrumentation through the working channel of the endoscope.

There were no intraoperative complications, with minor venous bleeding easily controlled through saline irrigation and bipolar coagulation. In no case was it necessary to convert the endoscopic procedure to craniotomy.

The procedure was well tolerated and safe for all patients. Postoperative and 3-month follow-up MRIs demonstrated complete removal of the cyst, including both the content and the capsule, in all patients, with reduction of ventriculomegaly. In 3 of 5 cases, MRI at 1-year follow-up showed complete removal of the cyst and regression of ventriculomegaly.

Immediate improvement in headache symptoms was observed in all patients, and those with cognitive or gait impairments experienced progressive recovery in the following months. The average length of hospital stay was 4 days (range 3–5).

A summary of all patient information is provided in Table 1.

**Table 1:** Patient information.

Patient	Sex	Age	Cyst size (mm)	Ventriculomegaly	Symptoms	Approach^a^
1	F	28	10.5	Yes	Headache, memory deficits, difficulty in concentrating	Left TF
2	F	35	12	Yes	Headache, nausea/vomiting	Right TF
3	F	43	15	Yes	Headache, nausea, impaired gait	Right TF
4	F	65	9	No	Headache	Left TF
5	F	48	13.5	Yes	Headache, nausea/vomiting	Right TF

^a^ TF, transforaminal.

### 3.1. Illustrative cases

#### 3.1.1. Case 1

A 28-year-old female patient presented to our outpatient clinic complaining of headache for approximately two years and, for the past six months, short-term memory deficits and difficulty keeping focused while working.

**Figure 3:**
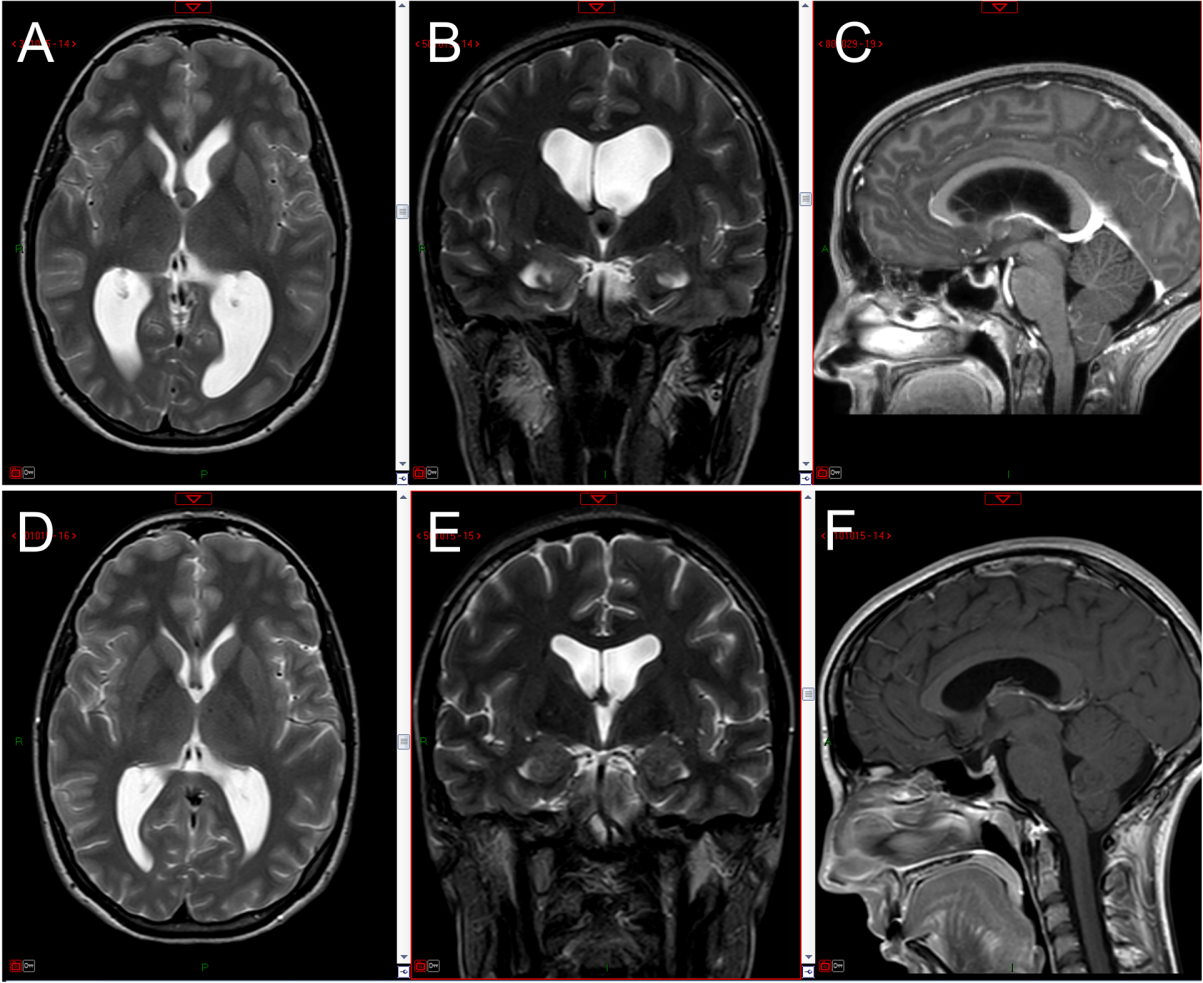
Preoperative MRI images (axial and coronal T2-weighted, sagittal enhanced T1 weighted) showing the third ventricle colloid cyst with bilateral (left > right) ventriculomegaly (**A–C**); three-month postoperative MRI images showing complete removal of the cyst with marked reduction in ventricular size (**D–F**).

Contrast-enhanced MRI revealed a 10.5 mm colloid cyst of the third ventricle, slightly deviated to the left of the midline, resulting in significant enlargement of the lateral ventricles, particularly on the left side (Figures 3A–C).

The patient underwent a minimally invasive endoscopic procedure with left frontal access for complete removal of the cyst, assisted by the Artemis Device (Supplementary Video 1). Postoperative MRI performed 48 h after surgery confirmed the successful removal of the cyst, as evidenced by the absence of any residual cystic components (Figures 3D–F).

This case represented our first experience using the Artemis Device for colloid cyst removal. As can be appreciated in the video, we were immediately able to assess the comfort and ease of use of this device in removing both the capsule and the mucinous contents in order to achieve a truly complete removal of the cyst.

The patient was discharged on the third postoperative day with complete resolution of her headache. At a three-month follow-up appointment, the patient reported significant improvement in her previously experienced memory and concentration deficits, leading to notable enhancements in her work performance.

#### 3.1.2. Case 2

A 43-year-old female patient presented to our clinic with a history of worsening headache over the past year, accompanied by nausea and unsteady gait.

**Figure 4:**
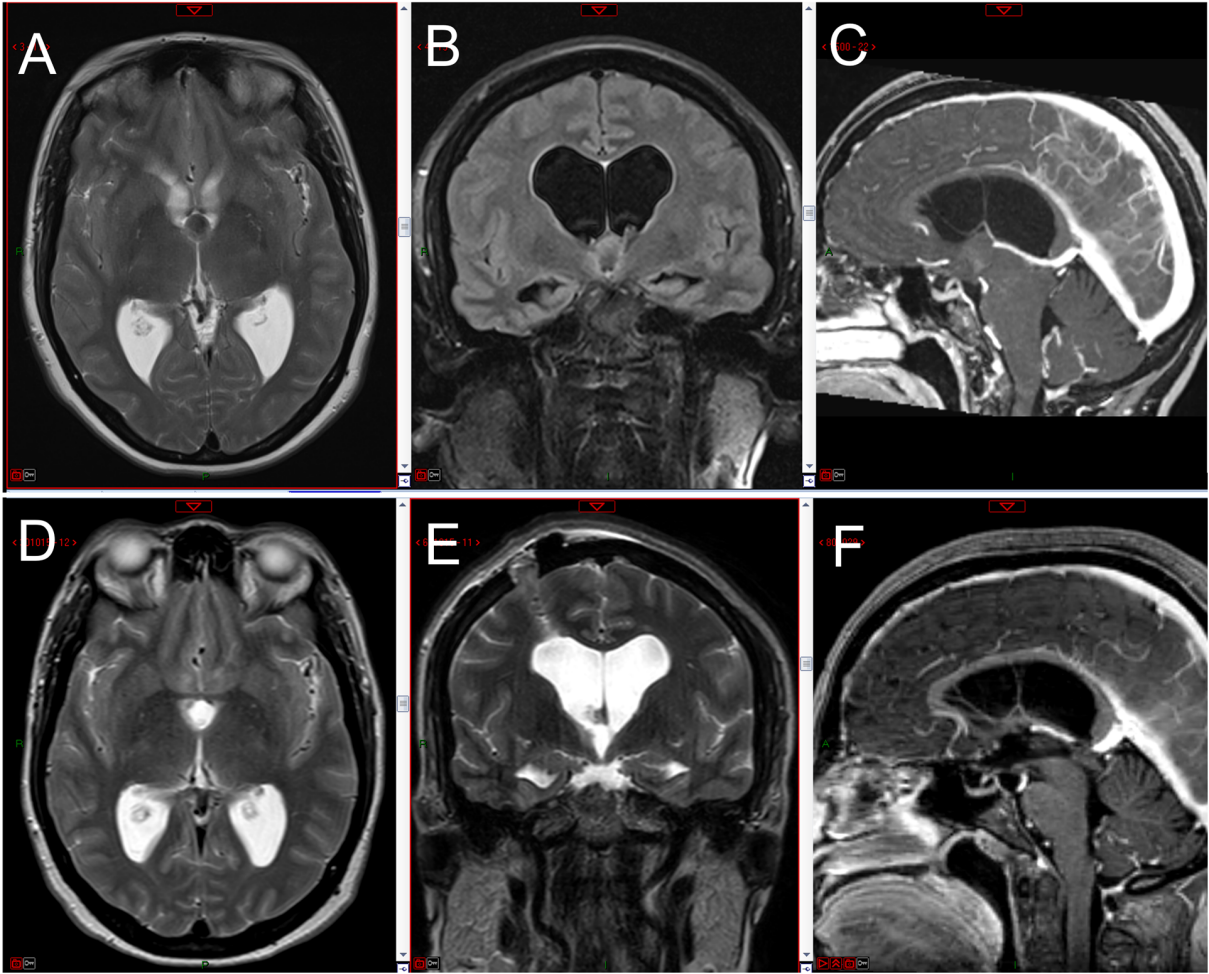
Preoperative MRI images (axial T2-weighted, coronal FLAIR T2-weighted, sagittal enhanced T1-weighted) showing the third ventricle colloid cyst with bilateral ventriculomegaly (**A–C**); 48 h postoperative MRI images showing complete removal of the cyst with initial reduction in ventricular size (**D–F**).

After neurologic consultation, she underwent a contrast-enhanced MRI showing a colloid cyst of the third ventricle of 15 mm in size, causing obstruction of both foramina of Monro and consequent hydrocephalus (Figures 4A–C).

The patient underwent cyst removal using a right frontal endoscopic approach assisted by the Artemis Device. On endoscopic view, the cyst was largely hidden below a narrow foramen of Monro and therefore removal was more challenging (Supplementary Video 2).

The video clearly depicts the unfavorable position of the cyst. In this case, the suction system provided by the Artemis allowed us to bring the cyst into a favorable position to be more easily fragmented and suctioned. Additionally, the device's adjustable suction power helped prevent damage to the thalamostriate vein, demonstrating its efficacy and safety.

Postoperative MRI confirmed complete removal of the cyst accompanied by an initial reduction in ventricular size (Figures 4D–F).

The patient was discharged on the third postoperative day with no headache. At the three-month outpatient follow-up, the patient reported complete recovery of walking stability.

## 4. Discussion

Innovation and technology in neurosurgery are advancing rapidly, leading to the introduction of a growing number of tools aimed at assisting surgeons and improving surgical outcomes.

By the development of appropriate tools, there has been great interest in pure endoscopic treatment of colloid cysts because of their midline and intraventricular location, cystic shape, and absence of major bleeding (5–9).

The goal of colloid cyst resection is the complete excision of the colloid cyst capsule and its contents to prevent future recurrence.

In the past, the most common approach for surgical resection of colloid cysts involved a craniotomy and microsurgical resection through a transcallosal, transcortical, or subfrontal approach. Studies on microsurgical colloid cyst resection have shown a high success rate in terms of cyst removal with low recurrence rates, thanks to the larger workspace, availability of surgical instrumentation, and bimanual surgical dissection (3, 4, 9).

Simple suction of the colloid without complete removal of the cyst wall often results in recurrence, at a rate up to 80% (5, 7, 9, 10).

However, the risks and complications associated with the resection of the colloid cyst wall should not be overlooked due to its close proximity to important deep venous tributaries, the fornices, and the hypothalamus (1). For this reason, a partial removal of the cyst wall, combined with coagulation of the insertion, can be considered an effective option when the cyst is firmly attached to the tela choroidea (10).

In this regard, for colloid cysts firmly attached to the tela choroidea or located in the middle-posterior third of the roof of the third ventricle, Iacoangeli et al. proposed a combined transforaminal-transchoroidal approach using scissors, bipolar coagulator, or a deflated Fogarty catheter as a dissector, exploiting both working channels of the rigid endoscope (11). Anyway, this approach has limitations due to the difficulty of bimanual endoscopic dissection, unlike microsurgery.

In alternative, Chibbaro et al. proposed an anterior trans-frontal endoscopic approach to reach the roof of the cyst and detach it from the tela choroidea more easily and safely. In this way, they stated a total cyst removal rate of 86.2% (12).

Rangel-Castilla suggested that an estimated entry point of 4 cm perpendicular to the midline and 4.5 cm anterior to the coronal suture can be used in patients with ventriculomegaly (13). For this anterior frontal approach, an intraoperative navigation should be considered.

Anyway, the anterior trans-frontal approach is criticized. Due to an angle of the foramen of Monro of about 20° to the sagittal plane, in order to gain complete visualization of the lesion, it is necessary to push the endoscope medially, resulting in compression of the anterior column of the fornix and the anterior septal vein (11).

The optimal outcomes in endoscopic removal of intraventricular lesions are influenced not only by the characteristics of the lesion itself, including its location, size, density, and vascularity, but also by the accessibility of advanced endoscopic devices.

The Artemis Neuro Evacuation Device is a low-profile aspiration and fragmentation system that was designed initially for intraventricular hemorrhage evacuation and later for deep-seated intraparenchymal hemorrhage evacuation. It has also been proposed for other conditions such as cerebellar hemorrhages, acute subdural and epidural hematomas, abscesses and pituitary adenomas (14–19). However, its therapeutic value in treating colloid cysts has not been explored. For such reason, our case report presents a novel application of the Artemis Device.

In 2016 Tan et al. described the advantages of the Apollo System (Penumbra, Alameda, California, USA) for the minimally invasive evacuation of intraventricular hemorrhages, which is a similar use of the device to ours (14). The Apollo Wand was then introduced into the ventricle via the working channel of the neuroendoscope and blood clot removal was achieved through a combination of aspiration, vibration, and irrigation. The results were favorable, with a significant reduction in blood clot volume in the accessible ventricles.

Based on our successful experience using the Artemis Device in five procedures for endoscopic removal of intraventricular hematomas, we have decided to expand its use for the removal of other intraventricular lesions, including colloid cysts. Our decision to utilize the Artemis Device for endoscopic removal of colloid cysts is based on our prior experience with the standard technique for removing these lesions.

In all our clinical cases, the cysts were not firmly attached to the tela choroidea or located in the middle or posterior third of the roof of the third ventricle, eliminating the need for a transchoroidal or anterior frontal approach.

In any case, if the cyst is not strongly attached to the tela choroidea, the controlled suction provided by the Artemis Device causes the cyst to detach from the superiorly placed tela and protrude into the Monro. If a small residual fragment remains, it can be visualized using a 30-degree endoscope and, if feasible, removed.

In fact, the Artemis Device allows the transchoroidal approach not to be performed except when the cyst is attached medio-posteriorly to the roof of the third ventricle.

Indeed, although using a good endoscopic surgical equipment, we noticed that with the use of standard endoscopic instruments, such as scissors, forceps, and mono- or bipolar coagulation systems, cyst removal was rather challenging and not always complete, both in terms of removing the capsule and mucinous content, also lacking a precise, safe and effective suction system. In this regard, Souweidane proposed a No. 6 French plastic endotracheal catheter with adjustable suction for the removal of solid intraventricular tumors, highlighting the advantages of transparency in distinguishing the tumor from other structures during aspiration (20). However, this device does not provide an effective fragmentation system combined with a controlled suction system for removing the solid component of the cyst.

Actually, this new technology allowed us to safely remove the cyst without “digging” beyond the cyst borders, thus avoiding without iatrogenic vascular or parenchymal damage.

By incorporating both aspiration and fragmentation functions into one device, the Artemis brings the bimanual advantages of microsurgery to endoscopic procedures. Additionally, compared to traditional endoscopic surgical techniques, this neuroevacuation device overcomes the limitations of piecemeal removal of the cyst's capsule and solid contents through its effective fragmentation system. Its equally effective suction function, equipped with a precise pressure control system, provides a viable alternative to other systems proposed by different authors, such as flexible nitinol stone retrieval baskets, endotracheal tubes, or central venous cannulas. Actually, the choice among these systems depends heavily on the surgeon's preference or the available equipment (21, 22).

As in brain tumor surgery, the use of aspirators and scissors has been gradually replaced by the advent of ultrasonic aspirators, we believe that the Artemis Device has the potential to play a crucial role in the endoscopic removal of colloid cysts, either as a standalone device or in combination with conventional instrumentation.

However, future multi-center studies are needed to further investigate its impact on long-term clinical outcomes.

## Data Availability

The original contributions presented in the study are included in the article/**Supplementary Material**, further inquiries can be directed to the corresponding author.

## Supplementary material

Supplementary Video S1 The video shows the first surgery we performed with the Artemis device for the removal of a colloid cyst. As you can appreciate, we were immediately able to assess the comfort and ease of use of this device in effectively removing both the cyst's capsule and its mucinous contents.

Supplementary Video S2 In the video, you can observe the unfavorable position of the cyst which was mostly concealed beneath a narrow foramen of Monro. However, thanks to the suction system offered by the Artemis device, we were able to reposition the cyst into a more favorable location, facilitating its fragmentation and suctioning.
